# The Radon Gas in Underground Buildings in Clay Soils. The Plaza Balmis Shelter as a Paradigm

**DOI:** 10.3390/ijerph15051004

**Published:** 2018-05-17

**Authors:** Carlos Rizo Maestre, Víctor Echarri Iribarren

**Affiliations:** Department of Building Construction, University of Alicante, Carretera San Vicente del Raspeig, s/n, 03690 San Vicente del Raspeig, Spain; victor.echarri@ua.es

**Keywords:** radon, underground building, construction materials, healthy architecture, environment, heritage construcion

## Abstract

In healthy buildings, it is considered essential to quantify air quality. One of the most fashionable indicators is radon gas. To determine the presence of this element, which is harmful to health, in the environment, the composition of the soil is studied. The presence of radon gas within a building depends both on the terrain in which it is located and on the composition of the materials of which it is composed, and not as was previously believed, only by the composition of the soil (whether granitic or not). Many countries are currently studying this phenomenon, including Spain where the building regulations regarding the accumulation of radon gas, do not list in their technical codes, the maximum dose that can a building can hold so that it is not harmful to people and the measures to correct excessive accumulation. Therefore, once the possible existence of radon in any underground building has been verified, regardless of the characteristics of the soil, the importance of defining and unifying the regulations on different levels of radon in all architectural constructions is evident. Medical and health science agencies, including the World Health Organization, consider that radon gas is a very harmful element for people. This element, in its gaseous state, is radioactive and it is present in almost soils in which buildings are implanted. Granitic type soils present higher levels of radon gas. Non-granitic soils have traditionally been considered to have very low radon levels. However, this paper demonstrates the relevant presence of radon in non-granitic soils, specifically in clayey soils, by providing the results of research carried out in the underground air raid shelter at Balmis Square in Alicante (Spain). The results of the measurements of radon accumulation in the Plaza Balmis shelter are five times higher than those obtained in a similar ungrounded building. This research addresses the constructive typology of an under-ground building and the radon presence in its interior obtained using rigorous measurement techniques.

## 1. Introduction

Radon gas is produced as a result of the decay of uranium contained in rocks [1]. Radon flows from the soil and is mostly concentrated in closed areas [2], so it is highly recommended that homes and workplaces are properly ventilated [3]. Three quarters of the radioactivity in the environment comes from natural elements [4]. Radon is the largest source of natural radioactivity [5] and the public health problem that its concentration generates both inside buildings and in drinking water makes it necessary to consider it for evaluation [6].

Radon decays due to so-called ionising radiation because when it penetrates matter, it usually pulls electrons from the surrounding atoms by a process known as ionisation [7]. If the matter is a biological structure with a high water content, the ionisation of water molecules can give rise to so-called free radicals with a high level of chemical activity, enough to alter important molecules that are part of the cells of living organisms [8]. These alterations may include chemical changes in DNA, the basic organic molecule that is part of the cells that make up our body [9]. These changes may lead to biological effects, including abnormal cell development [10]. These alterations can be more or less severe depending on the dose of radiation received [11]. The main effect of the presence of radon in the human environment is the risk of lung cancer [12]. The presence of people inside a building helps the movement of air and therefore the renovation. However, if the accumulation values are high, additional corrective actions should be taken. This radioactive gaseous element is present in almost all construction materials, and in the soil where buildings are implanted [13,14]. There are different radon measuring devices. Some are active, require electricity and allow a continuous record of the concentration and fluctuations of radon gas during the measurement period [15]. Others are passive and do not require electric power to operate in the sampling environment [16]. Ionic Chambers of Electretes (ICE) have been used to carry out this research. ICEs are passive devices that function as integrative detectors to measure the average radon gas concentration during the measurement period. The electret functions both as a generator of an electric field and as a sensor in the ion chamber. The radon gas enters the chamber by diffusion through an inlet equipped with a filter without allowing the rest of the elements produced during the decay process to pass through [17]. Radiation emitted by radon, and its decay products formed inside the chamber, ionise the air contained inside the chamber reducing the voltage of the detector surface [18]. Subsequently, a calibration factor relates this voltage drop to the concentration of radon in the studied space and time.

In isolated constructions, or on the ground floor of buildings, the most important source of radon is the ground. The radio concentration in the ground is generally between 10 and 50 Bq/m${}^{3}$, although it can reach much higher values. The average value is around 40 Bq/m${}^{3}$. The amount of radon that enters an interior from the ground depends mainly on the concentration of radium-226 in the subsoil and the permeability of the subsoil.

In most countries there are predictive maps of radon content, mainly representing the igneous composition of the soil [19,20,21,22] For example, Sweden has developed maps based on the measurement of the geogenic potential of radon, which indicate the risk level by zone, estimated from the concentration of radon in the ground at 1 m depth [23,24,25]. Likewise, the usefulness of methods based on other variables, such as soil radio-226 concentration or uranium equivalent has also been tested. In the case of France, for example, the national map has been compiled from geological maps and average uranium content of each geological unit [25,26,27]. The German map [28] and the Czech map [29] have also been developed using the geogenic potential of radon. All the radon gas predictive maps consider that granitic soils are the most risky in terms of their concentrations. They consider clayey soils as having a low presence of radon gas. In Spain, in February 2018, the Technical Building Code (CTE), does not yet state the dose of radon that one building can contain at most and how to control it [30,31].

The soil of the Spanish Mediterranean coast, where the city of Alicante is located, is mainly clayey [32]. The underground air raid shelter of Seneca Square is located in the city centre of Alicante (Spain). This shelter was built in 1937 during the Spanish Civil War (1936–39), and was restored in 2011 in order to be part of the city’s museum tours. Different aspects of the shelter have been studied: the constructive typology of the underground building, its historical context and the presence of radon on the inside obtained through rigorous measurement techniques. By virtue of the results obtained from the measurements, the shelter serves as a paradigm to demonstrate the relevant presence of more than 100 Bq/m${}^{3}$ of radon in the interior of an underground building in non-granitic soil [22].

## 2. The Plaza Balmis Shelter. Construction and Restoration

The Spanish Civil War took place in Spain between 1936 and 1939, where two sides, the Republicans and the rebels fought to take control of Spain [33]. The bomb shelters of the Civil War in Alicante, are a singular type of architectural construction that conserves little information because they were developed without technical documentation conditioned by the urgent need to build shelters to protect the population [34]. From the documents present in the Municipal Archive of Alicante (MAA), it is known that, in 1937, there were 18 shelters with capacity for 8070 people and 15 more were to be built for 9900 people. In 1938, there were 55 shelters with a capacity for 38,140 people and 37 more shelters were planned that would have provided the city with a total capacity for 108,590 people. At present, there are about 90 shelters that were built during the war.

**Figure 1:**
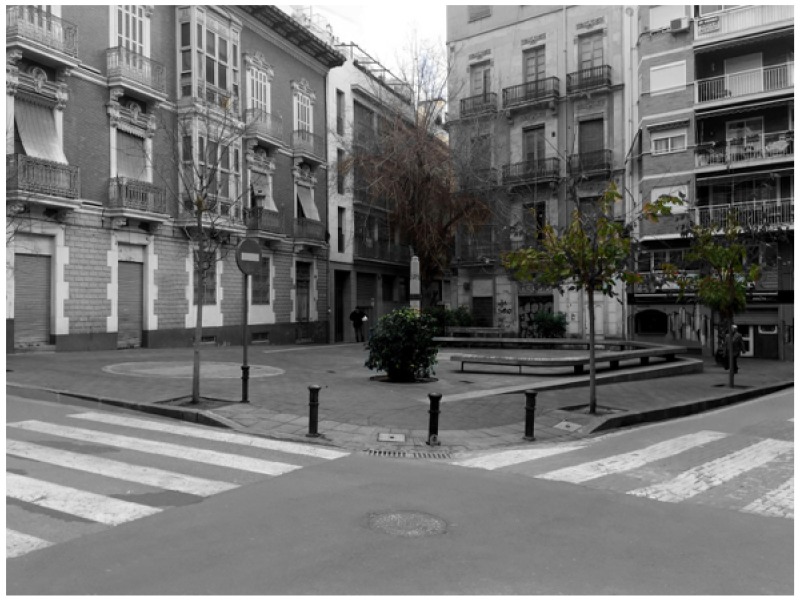
Doctor Balmis Square at present.

The shelter at Doctor Balmis Square is located in the square that bears his name, Figure 1. Shelter R46, as it is known according to the authorities currently responsible for the conservation of the city of Alicante, has two accesses: one on Canalejas street and the other, at the intersection of Limones and El Cid streets. The second access is walled up, probably since the demolition of the roofs carried out by the government since 1945.

The bombardments of 1939 were devastating for the area of the square and the surroundings areas due to the siege of the city by the rebel side. In September 1945, six years after the end of the war and in the middle of Franco’s dictatorship, a project was drawn up for the demolition of all the constructions on the ground floor of the streets or squares, entailing the demolition of the roofs of the shelters with these characteristics. The decision to dismantle the roofs of this type of buildings was provoked by the complaints of the neighbours due to their dilapidated state, the bad smells caused by humidity and the beginnings of deterioration by detachment.

In the years after the war, during the Franco dictatorship, the square received different treatments, and renovations were carried out that culminated in the development of the shelter by the City Council of Alicante for visitors who were interested in the history of the city in the Spanish Civil War.

**Figure 2:**
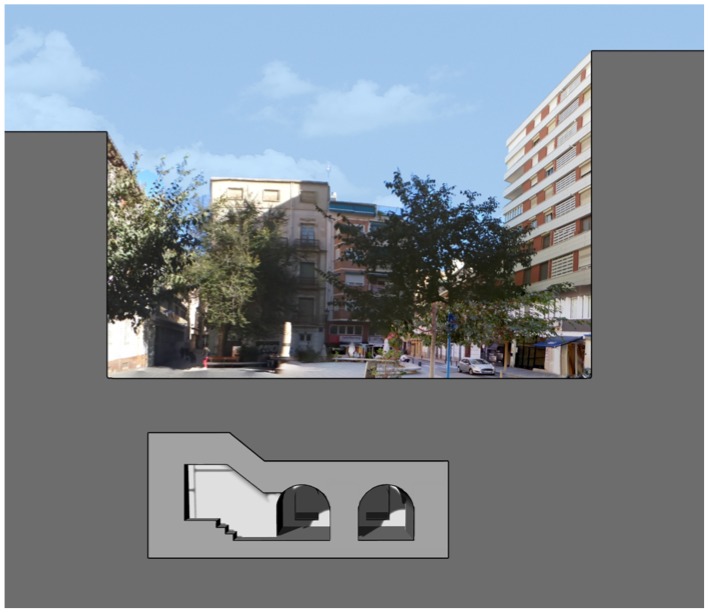
Photomontage of the Plaza Balmis section in its current state.

**Figure 3:**
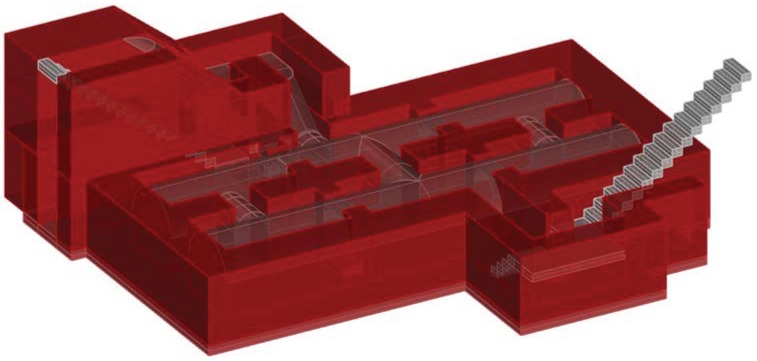
Image of the volumetry of the refuge of Plaza Balmis where the two entrances are shown. The right corresponds to that of the streets Limones and Cid, currently walled and the left corresponds to the only entrance available. The vaulted roofs are shown in a lowered semicircular arch. In the upper part there is a large concrete layer of up to 1.5 m, this side being the most protected.

The shelter at Plaza Balmis is cellular, type 3, according to the characteristics of the municipal architect of the time, the shelter’s body being buried two meters below the level of the square (Figure 2). The volumetric layout of the shelter (Figure 3) is composed of six rectangular cells with a surface area of approximately 7.5 m${}^{2}$ in each one of them with a height ranging from 1.81 m to 2.33 m. The six rooms are connected by passages of approximately 1 m. The shelter complex has about 50 m${}^{2}$ without accesses. The two central partitions are connected to the accesses and serve as a distribution for the other four that consisted of a stone bench. The galleries are separated by walls of 0.60 m width, made of cement mortar masonry and plaster up to half height. The lateral chambers are separated from the two central chambers by rings that brace the structure in its lower part and reinforce the upper part with semi-circular arches lower than those used in the vaults.

**Figure 4:**
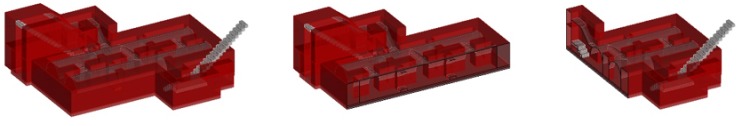
3D reconstruction images of the refuge, showing cross sections in both directions.

**Figure 5:**
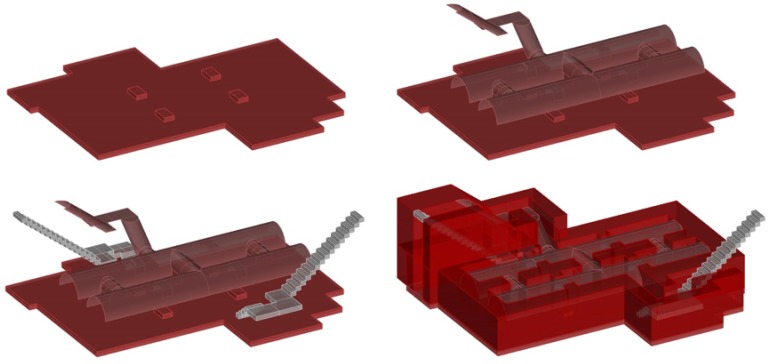
Images of the reconstruction in 3D of the refuge of Plaza Balmis, showing the two longitudinal naves that form three quarters each connected in both directions.

The construction, as shown in the Figure 4 and Figure 5 has two zigzag accesses at the end of each stairway, to attenuate the effects of the shock waves caused by the bombs and in the event that one of them was not usable, the other was the escape route for the occupants of the shelter. The entrances are built on a base of mass concrete that serves as a support for the stairways built with hollow brick. The interior access to the square, corresponding to the intersections of Limones and Cid streets, is currently covered due to different developments that have been carried out in the square. The walled access was probably filled with rubble from the roof demolition carried out in 1946.

**Figure 6:**
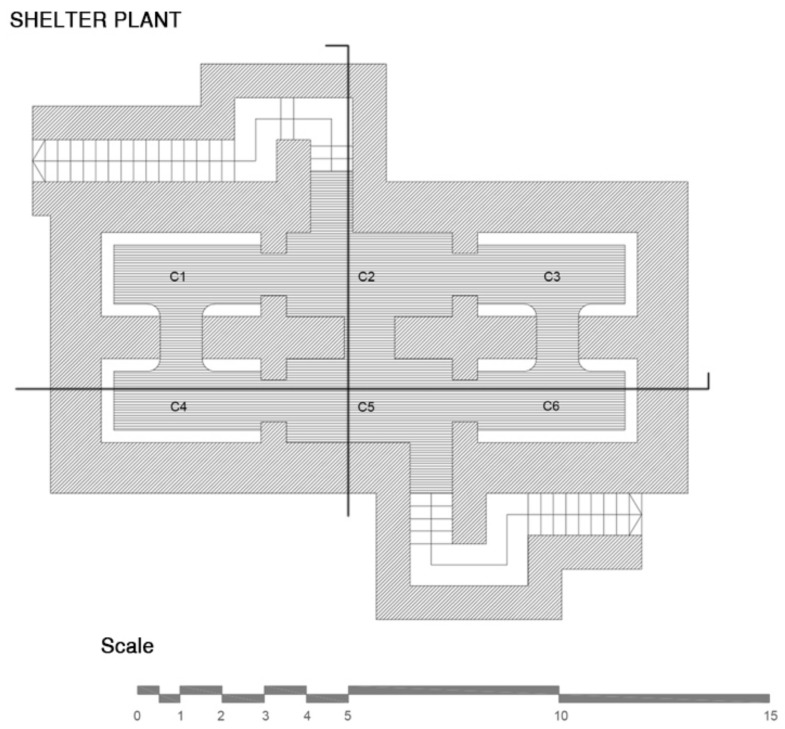
Plant of the refuge of Plaza Balmis with six cells connected in both directions forming two large longitudinal halls.

**Figure 7:**
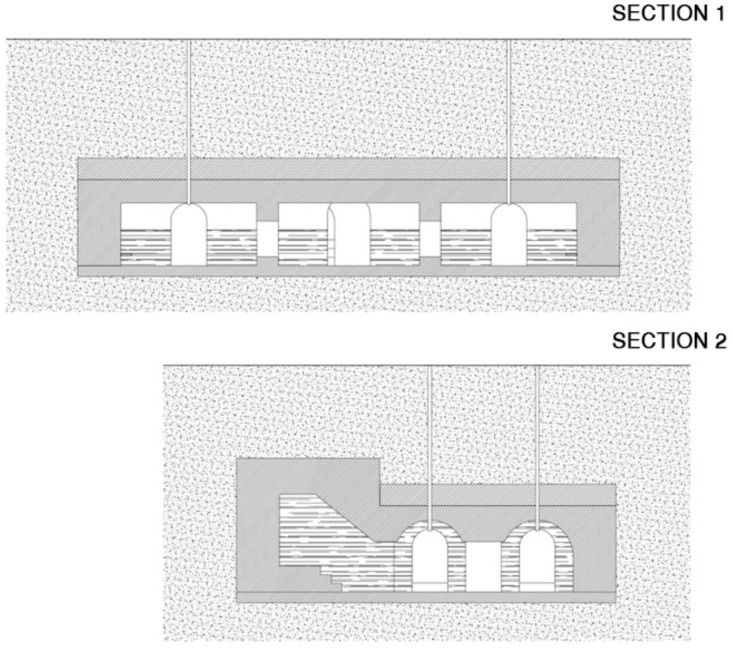
Section of the Plaza Balmis shelter with the three corridors connecting the three spaces that divide the two naves.

The upper enclosure is made of hollow brick, placed in the form of barrel vaults with lowered arches, ready for tambourine and plastered as shown in the sections in Figure 6 and Figure 7. The upper top of the vaults is made of reinforced concrete, the pavement is made of mass concrete filled with compacted sand. In the original layout of the shelter, the roof contained the doorways to the enclosure and was the only direct contact to the outside, thus achieving greater protection.

The ventilation of the whole structure was through 20 vents placed on the longitudinal axis of the vaults of the galleries. It is a set of ceramic pipes that go from the top of the roof of the shelter to the old roof ridge , which has disappeared. The base of each vent was covered with a layer of plaster that has been almost completely lost.

**Figure 8:**
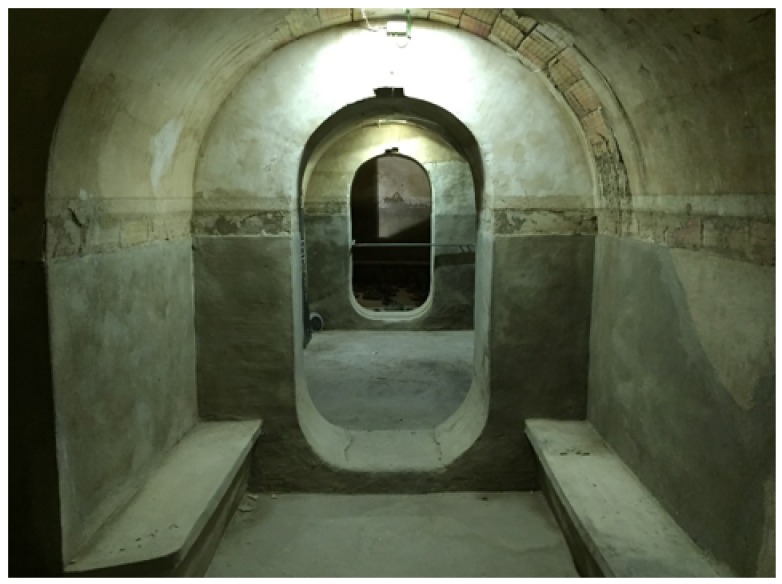
One of the two main halls, where the three cells of this one are distinguished, being the distribution center.

At present, the shelter has deteriorated due to the high humidity present in the interior (Figure 8) generated by the upper square and the poor ventilation, produced by the only available access.

The square has had many modifications since the shelter was inaugurated in 1938. In 1945, with the Second World War ended and with the passing of the fear of possible attacks on the city, it was decided to demolish the roof for its deterioration, this being the only intervention in its original structure. This public space is located in the centre of Alicante, and despite its small size, has great importance from the 21st century onwards because of its value as a historical route. The surrounding buildings also have great heritage value for the city. In the past it was recognised for its modernist style, represented on the perimeter bench that ran along it, with colourful tiles reminiscent of the work of the Catalan architect Antonio Gaudí.

**Figure 9:**
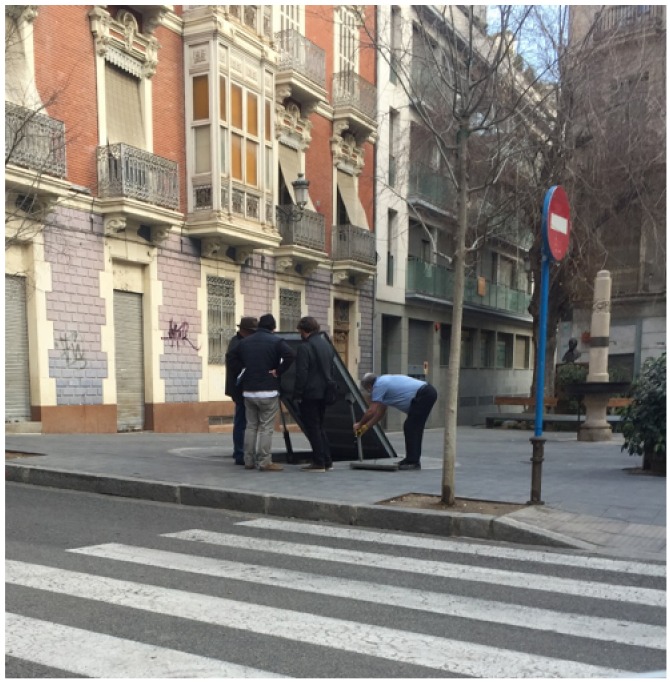
Image showing the opening of the hydraulic access to the Plaza Balmis refuge on Canalejas Street. This entry is being modified by a fixed type for greater security.

In 2007, the City Council of Alicante plans to remodel the square space in order to modernise it. This is great resistance among the inhabitants and the traders because they consider that the square is in good condition and that there are more important areas needing improvement. For this reason, the modernisation of the square was halted up to 2013 when the project was restarted. During the course of the work, the shelter appears and generates great expectation among the population. Finally, it was decided to respect and value it, and to add an access from Canalejas Street, which is integrated into the pavement and opens with hydraulic elements as seen in the Figure 9.

The building, being underground and poorly ventilated, makes the ground where it is inserted into its material composition. Therefore, the formal composition of the building is not only the different densities concrete with which it was built. The mineralogical composition of the soil means that, although there is little presence of possible radon precursors, this element accumulates inside the building.

## 3. Methodology for Measuring Radon Gas in the Shelter

**Figure 10:**
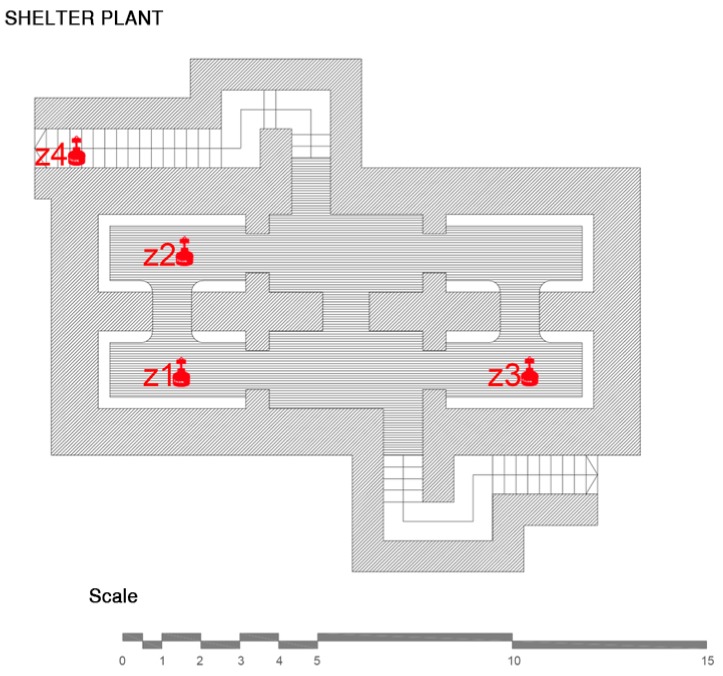
Arrangement of the zones chosen for measurements at the Plaza Balmis refuge.

The data collection of the shelter of the Plaza del Doctor Balmis was carried out over 22 days, from the 15th of March 2016, and it took place homogeneously throughout the shelter. The measurement zones of the study correspond to the cells, as shown in Table 1 and Figure 10.

The plan of the shelter, with its absence of complete partitions, allows all rooms to be connected and to act in a homogeneous way, with ventilation only produced from the access that is available. The walled access generates a bottleneck where the air does not regenerate as much as in the other parts of the shelter. There are also two grilles on the old vents, but they have collapsed and they cause moisture problems.

The radon gas metering system used in the study over the 21 days was the short chamber and short electret of the Eperm System. The short-chamber system is used to monitor radon gas buildup in a short period of time. The measuring tool counts the voltage drop in a charged element, as the presence of radon gas favours the discharge. This discharge is then analyzed in the laboratory and related to the space and time of measurement. The plant typology of the site means that all rooms are connected, which makes it possible to foresee that the quantities of radon gas are similar in all of them. The short-short type of camera-electrete measurement is recommended for measuring between 5 and 7 days, but in this case it was impossible to enter the shelter after this time due to the restrictions of those responsible for guarding it. However, the results were not influenced by the longer duration of the measurement as indicated by the specifications of the system used. The climatology during the study was as follows: Rainfall only occurred on 21 March, 4 and 5 April, with 3 mm accumulated between the three days, which did not greatly affect the ionisation of the site and therefore the rise in the presence of radon gas. Temperatures during the 22 days of the study fluctuated between 26 °C maximum and 7 °C minimum reached during the early morning of March 16. Inside the shelter the temperature is usually kept constant, around 16 °C.

## 4. Results Analysis

The results obtained in the different measurements are compared with the 100 Bq/m${}^{3}$ and 300 Bq/m${}^{3}$ recommended by the European directive 2013/59/EURATOM of the year 2014 suggested as maximum recommended by the European Commission for Atomic Energy [35] . Therefore, 100 Bq/m${}^{3}$ is considered as the first threshold from which permanent monitoring of the gas, presence measurement and the level of 300 Bq/m${}^{3}$ are considered as the threshold from which, mandatory ventilation increase measures must be taken.

The equipment introduced into the Plaza Balmis shelter was placed for 22 days in the different rooms with a unique combination of camera-electrete and short-short. For the representation of the results, a summary Table 2 shows the different measurement zones and the values of the mean environmental concentration obtained from radon gas, in Bq/m${}^{3}$.

In the shelter, there is a disparity in the results; in Zone 3, 96.98 Bq/m${}^{3}$ and in Zone 4, corresponding to the secondary entrance, 224.67 Bq/m${}^{3}$ are reached, that is, more than double the value according to the stay, although both are communicated. This is caused by the distance from each place to the entrance and the different currents generated inside. The main access and the two grilles of the old vents renew the air in the main nucleus of the shelter, but the area of the entrance that is covered with walls does not have such a continuous air supply, an aspect that appears in other similar actions analysed by other authors such as Martín Matarranz or Juan José Llerena [36,37].

Based on the results obtained during the study at the Plaza Balmis shelter, a comparison is made in the different places measured with short chamber and short electrode. As shown in Table 2, the radon gas values in the air reach 224.67 Bq/m${}^{3}$ at the secondary input (Zone 4 of the measurement).

**Figure 11:**
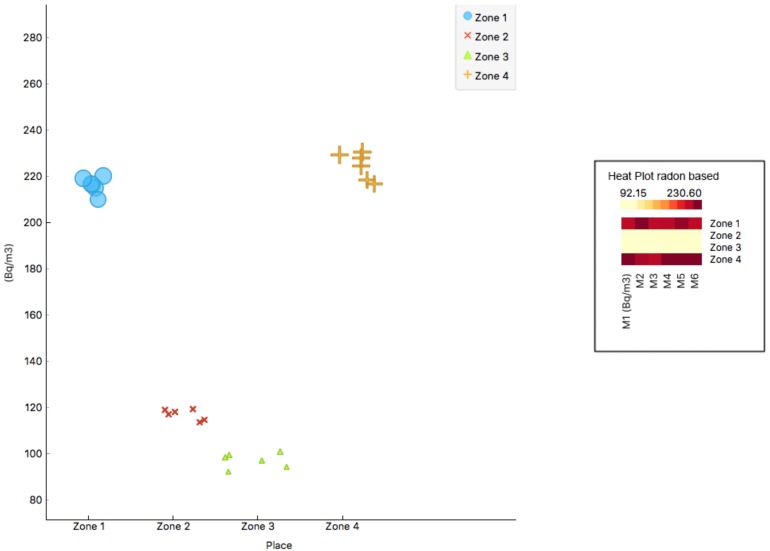
Scatte Plot. Symbol size proportiona and Heat Plot, the different measurements in the coloured areas depending on the presence of radon obtained in each of theml.

In the views of the analysed data displayed in the Figure 11, the values obtained in the different measurement zones are below the threshold of 300 Bq/m${}^{3}$, established within this study as a value above which it is necessary to take corrective ventilation measures. Zones 1 and 4 are the only ones above 100 Bq/m${}^{3}$, a value above which the presence of radon gas in buildings used by people must be monitored.

**Figure 12:**
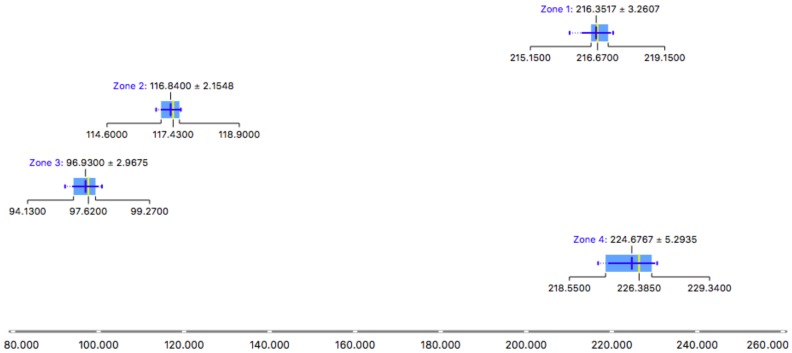
Box Plot zones measures on Plaza Balmis Shelter.

**Figure 13:**
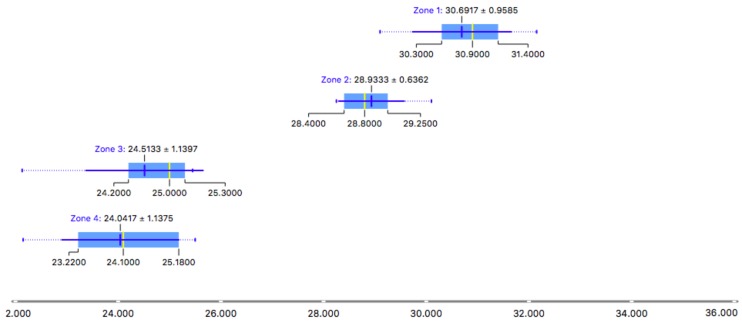
Box Plot zones measures on unburied building.

As it can be seen in the results representation Figure 12 all measurements are above 100 Bq/m${}^{3}$. The values of zones 1 and 4 are particularly relevant, with values close to 220 Bq/m${}^{3}$. The purpose of this research article is to demonstrate the hypothesis that the accumulation of radon gas in buried buildings is greater than that which can occur in unburied buildings, even on clay soils. Clay soils have traditionally been considered of low risk for accumulation of radon. In the early phases of the study and to test the results, measurements were made in unburied defensive buildings, built in concrete and from the same period as the Balmis shelter. In one of these constructions, 24 measurements were taken in four different zones. The results are shown in Figure 13. These values serve as a comparison of the two constructions, since they are above 25 Bq/m${}^{3}$, in constrast with values higher than 220 Bq/m${}^{3}$. The shelter of Plaza Balmis presents values five times higher than the similar one not buried, therefore, the non-granitic lands also favour the radon gas accumulation.

## 5. Conclusions

The Shelter of Balmis Square of Alicante (Spain) is a defence shelter building was used as a paradigm to demonstrate the presence of radon gas in underground constructions located in clay soils (non-granitic) that are usually considered low risk in terms of the potential presence of radon gas.

The southern Mediterranean coast where Alicante is located is considered a low or very low risk area within the Spanish map of natural radiation (MARNA). This paper has studied the architectural details of the shelter, located in the Balmis Square of Alicante, details not currently available but the City Council intends to incorporate then into the museum’s history of the city.

The character of this historical construction, buried under a square in the centre of Alicante, with one of the entrances boarded up, makes for low ventilation inside the shelter. The renewal of produced air is generated only by the available access and by the small grilles of the old vents that are currently collapsed.

The indoor air quality inside the shelter has been studied, taking radon as a very important element to be diagnosed. This allows us to check whether in buildings with old building methods and that are very close to the ground, such as shelters, in this case with little ventilation, radon accumulations are produced.

The compilation of results has been evaluated with the Directive 90/143/EURATOM, where the European Union recommends a target radon gas level in the design phase of 100 Bq/m${}^{3}$ maximum for newly built buildings and an immediate level of action from 300 Bq/m${}^{3}$ for interior spaces from which remedial measures should be carried out in existing buildings. Although these values should not be taken as safe when they are less than 100 Bq/m${}^{3}$, they can be interpreted as a level from which to start paying attention and trying to minimise the values obtained by incorporating a more efficient form of air renewal and establishing more effective constructive methods to accumulate less radon gas.

The shelter forms a uniform volume, with all the cells connected and a single direct air inlet from the outside, coming from the access that is currently in service. With these conditioning factors, the values obtained oscillate between 96.98 Bq/m${}^{3}$ and 224.67 Bq/m${}^{3}$ at the walled entrance. These variations are explained by the different flows within the shelter. Only follow-up measurements of the amount of radon present should be taken for this installation, since all measurements are below the established 300 Bq/m${}^{3}$3 and almost all measurements are above 100 Bq/m${}^{3}$. At present, the shelter is unused and these results must be taken into account in the future, when it is opened to the public. Then, the movement inside will be greater air renewal will be improved.

The predictive maps must be concretised in their application by means of technical building codes, but in most countries, including Spain, the Technical Building Code (CTE) still does not specify the dose of radon that can be contained at most in a building and how to eliminate it.

The research community has already demonstrated that the radon gas is harmful to human health and has become a highly carcinogenic element and therefore, new regulations must incorporate this aspect as a control element. In view of the results presented in this article, it is clear that it is essential that there is compliance with measures (constructive, ventilation, etc.) to limit the presence of radon gas in buildings, especially in enclosed spaces used by people. Radiological studies on exposures to radon in underground workplaces and leisure areas (including public car parks, mines, subways, museums, tourist caves, etc.) should be compulsory in all cases. All this is regardless of the type of soil on which the buildings are built and the type of materials used.

## Figures and Tables

**Table 1:** Association of measurement zones and corresponding cell.

Zone	Cell
Zone 1	Cell 4
Zone 2	Cell 1
Zone 3	Cell 3
Zone 4	Secondary entrance walled

**Table 2:** Summary mean values of radon concentrations in zones.

Zone	Placement	Samples	Mean Radon Concentration (Bq/m${}^{3}$)
Zone 1	Cell 4	6	216.44
Zone 2	Cell 2	6	116.84
Zone 3	Cell 3	6	96.98
Zone 4	Secondary entrance walled	6	224.67
